# Bridging the knowledge-practice gap in chronic disease management: tele-education in diabetes care training

**DOI:** 10.20945/2359-4292-2026-0042

**Published:** 2026-04-20

**Authors:** Milena Coelho Fernandes Caldato

**Affiliations:** 1 Serviço de Endocrinologia e Metabologia, Centro Universitário do Pará, Belém, PA, Brasil; 2 Universidade do Estado do Pará, Belém, PA, Brasil; 3 Comissão de Formação Médica em Endocrinologia e Metabologia, Sociedade Brasileira de Endocrinologia e Metabologia, Rio de Janeiro, RJ, Brasil

The gap between knowledge acquisition and its translation into clinical practice remains a central and persistent challenge in chronic disease management ^(1)^. Nowhere is this more evident than in conditions such as diabetes mellitus, where effective care depends not on isolated decisions, but on longitudinal clinical reasoning, continuous therapeutic adjustment, and sustained patient engagement over time. Despite advances in medical education, training environments continue to privilege acute, high-intensity care, often at the expense of competencies required for managing chronic illness ^(2,3)^.

In this issue, the study by Lopes and cols. ^(4)^ provides timely and relevant insights into this problem by evaluating a tele-education strategy implemented during residency training. Using a widely accessible digital communication platform, the intervention demonstrated a significant improvement in knowledge acquisition among residents. These findings reinforce the growing role of tele-education as a scalable and pragmatic tool to support learning, particularly in resource-constrained or high-demand clinical settings ^(5,6)^.

However, the study also highlights a critical limitation that has long been recognized in medical education: the persistence of the knowledge-practice gap. Despite robust cognitive gains, changes in clinical practice, as reflected by insulin management indicators, were modest and did not reach statistical significance ^(4)^. This disconnect suggests that clinical behavior is not readily modified by knowledge acquisition alone. Rather, it is shaped by a complex interplay of factors, including established prescribing habits, clinical culture, supervision structures, and system-level constraints ^(7-9)^.

Importantly, the absence of short-term improvements in patient-centered outcomes, such as length of hospital stay, should not be interpreted as a failure of the intervention. Instead, it reflects the inherent complexity of healthcare systems, in which outcomes emerge from multiple interacting determinants beyond individual physician knowledge. In this context, the apparent paradox, improved knowledge without measurable outcome change, becomes not only understandable, but expected within complex adaptive systems ^(10,11)^.

These findings invite a broader reflection on the limitations of current approaches to competency-based medical education. While knowledge acquisition remains a fundamental component of training, it is insufficient to drive meaningful changes in clinical practice when implemented in isolation. The management of chronic diseases requires competencies that are longitudinal, relational, and context-dependent-dimensions that are often underrepresented in traditional training models, which continue to prioritize immediacy, procedural skills, and acute care efficiency ^(1,2)^.

Within this evolving landscape, tele-education represents a promising, yet still under-integrated, strategy. Digital platforms-particularly widely accessible messaging applications-enable new forms of interaction that extend learning beyond formal clinical encounters, supporting case-based discussion, asynchronous feedback, and longitudinal mentorship ^(12,13)^. As demonstrated by the present study ^(4)^, these approaches are effective in enhancing knowledge. However, their capacity to influence clinical practice depends on their integration into structured educational ecosystems that incorporate supervision, audit and feedback mechanisms, and alignment with care processes.

Rather than functioning merely as adjuncts to traditional teaching, tele-education strategies should be understood as catalysts for reconfiguring how learning occurs in clinical environments-continuous, context-sensitive, and embedded within real-world practice. Bridging the knowledge-practice gap will therefore require not only the adoption of innovative educational tools, but also a deliberate effort to align educational strategies with the organizational and cultural realities of healthcare systems ^(12,13)^.

Ultimately, the contribution of this study lies in advancing a more nuanced understanding of the relationship between knowledge, practice, and outcomes in medical training. It reinforces a critical message for educators and institutions alike: improving what residents know is necessary, but not sufficient. Transforming clinical practice requires educational approaches that are longitudinal, integrated, and responsive to the complexity of real-world care ^(14,15)^.

## Data Availability

Datasets related to this article will be available upon reasonable request to the corresponding author.
